# Interpretable prediction of Ki-67 expression in breast cancer through integrated photoacoustic radiomics and clinical parameters

**DOI:** 10.3389/fonc.2026.1832391

**Published:** 2026-04-29

**Authors:** Mengyun Wang, Sijie Mo, Hui Shi, Zhibin Huang, Guoqiu Li, Jingjing Li, Qinghua Liu, Jinfeng Xu, Fajin Dong

**Affiliations:** 1 Department of Ultrasound, Shenzhen People’s Hospital, The Second Clinical Medical College, Jinan University, Shenzhen, Guangdong, China; 2 Department of Medical Ultrasound, Shanghai Tenth People's Hospital, School of Medicine, Tongji University, Shanghai, China; 3 Department of Ultrasound, Rizhao People’s Hospital, Rizhao, China

**Keywords:** breast cancer, Ki-67, photoacoustic, prediction, radiomics

## Abstract

**Purpose:**

This study aimed to develop a noninvasive method for preoperatively predicting Ki-67 expression in breast cancer (BC) by integrating radiomics features derived from photoacoustic/ultrasound (PA/US) imaging with clinical factors.

**Materials and methods:**

A total of 223 patients with pathologically confirmed BC underwent PA/US imaging before surgery. Radiomics features were extracted from tumor regions, standardized, and selected using statistical testing, correlation analysis, and least absolute shrinkage and selection operator (LASSO) regression. Ten machine learning algorithms were compared, and the best-performing classifier was used to construct the radiomics model. Clinical variables significantly associated with Ki-67 expression were then incorporated to build a combined model. Model performance was evaluated using receiver operating characteristic (ROC) curve analysis, calibration analysis, and decision curve analysis.

**Results:**

In the test set, the clinical, radiomics, and combined models achieved areas under the ROC curve (AUCs) of 0.722, 0.826, and 0.849, respectively. The combined model also demonstrated higher sensitivity (0.957) and negative predictive value (0.867), indicating strong capability in identifying patients with high Ki-67 expression. SHAP analysis confirmed that texture- and intensity-related imaging features played important roles in model prediction.

**Conclusion:**

PA/US-based radiomics provides a quantitative, interpretable, and clinically valuable approach for assessing tumor proliferation in BC. The combined model exhibited superior accuracy over single-modality models and shows promise as an effective supplementary tool for individualized preoperative evaluation of Ki-67 expression.

## 1 Introduction

Breast cancer (BC) is the most common malignancy among women worldwide and remains a leading cause of cancer-related morbidity and mortality (1). As a key indicator of cellular proliferation, Ki-67 plays an essential role in determining BC subtypes and guiding therapeutic decisions (2, 3). Elevated Ki-67 expression generally reflects more aggressive tumor behavior and poorer prognosis, whereas a decline after treatment often suggests better therapeutic response (4). Clinically, Ki-67 status is typically determined by immunohistochemical analysis of biopsy or surgical tissue. However, these procedures are invasive, time-consuming, and vulnerable to sampling bias due to intratumoral heterogeneity, which may lead to inconsistent assessments (5). In addition, for patients undergoing neoadjuvant therapy, Ki-67 levels can change substantially during treatment, yet repeated biopsies are usually impractical (6). These limitations highlight the need for a noninvasive and reliable approach to evaluate Ki-67 expression and to better capture the biological features of the entire tumor.

Currently, mammography, magnetic resonance imaging (MRI), and ultrasound are the most widely used imaging modalities for BC assessment. Mammography remains the primary tool for population screening and is effective for detecting microcalcifications, yet its sensitivity declines markedly in women with dense breast tissue, reducing diagnostic accuracy (7). MRI provides excellent soft-tissue contrast and valuable functional information through multiparametric imaging, helping delineate tumor extent and treatment response (8). Nevertheless, its high cost, prolonged examination time, and patient-specific contraindications restrict both accessibility and feasibility for longitudinal monitoring (9). Ultrasound, in contrast, is convenient, radiation-free, cost-effective, and widely used for routine breast evaluation. However, conventional ultrasound relies heavily on morphological features, which often overlap among breast lesions and limit diagnostic specificity while failing to reflect underlying biological heterogeneity (10). These limitations underscore the need for innovative imaging approaches capable of integrating structural, functional, and molecular information to better characterize tumor biology and guide personalized management.

The integration of photoacoustic (PA) imaging with ultrasound (US) has introduced a synergistic Multimodal PA/US technique that addresses several challenges in breast lesion evaluation. By combining the anatomical detail provided by US with functional and hemodynamic information obtained from PA imaging, PA/US enables real-time visualization of tumor morphology together with physiological parameters related to angiogenesis and metabolic activity (11). Through quantification of oxygenated and deoxygenated hemoglobin signals, PA/US offers a noninvasive means to assess tumor vascularity and tissue oxygenation, thereby enriching the understanding of tumor growth and metabolic behavior (12, 13). Previous studies have demonstrated that PA/US outperforms conventional US in classifying breast lesions and provides additional functional insights such as tissue oxygenation and blood-flow characteristics (14, 15). By concurrently capturing anatomical and functional features, PA/US shows significant potential for improving tumor characterization and supporting individualized assessment based on imaging-derived biomarkers.

Although PA/US imaging excels at visualizing both structural and functional features of breast lesions, its diagnostic performance still relies heavily on human interpretation. The complexity and volume of PA/US images can introduce observer variability, particularly in large-scale or longitudinal assessments (16). This underscores the importance of objective and quantitative analytic tools capable of reducing subjectivity and enhancing reproducibility. Radiomics offers a promising solution by transforming medical images into high-dimensional quantitative descriptors that reflect underlying tissue characteristics (17). Through automated feature extraction and statistical modeling, radiomics can uncover subtle imaging patterns that are imperceptible to human observers, enabling a more comprehensive and reproducible evaluation of tumor heterogeneity (18). Prior studies have shown the value of radiomics in BC classification, molecular subtype prediction, and treatment response assessment (19–21).

Building on these advantages, the application of radiomics to PA/US imaging is still in its early stage, yet emerging studies have already demonstrated its feasibility in BC, including distinguishing benign from malignant lesions and predicting molecular subtypes (22, 23). However, the relationship between PA/US-derived radiomic features and Ki-67 expression remains largely unexplored. Given that Ki-67 is a critical marker of tumor aggressiveness and therapeutic response, investigating its association with PA/US radiomics holds clear clinical relevance. Such an approach may offer a noninvasive, quantitative, and reproducible strategy for characterizing tumor biology and ultimately support individualized treatment planning and prognostic evaluation. Building on this rationale, the present study aimed to explore the potential of integrating PA/US imaging with radiomics to noninvasively predict Ki-67 expression in BC.

## 2 Materials and methods

This study followed the ethical standards of the Declaration of Helsinki and was approved by the institutional ethics committee (SYL-202161-02). All participants were fully informed of the study purpose and procedures and provided written informed consent.

### 2.1 Patients

**Figure 1:**
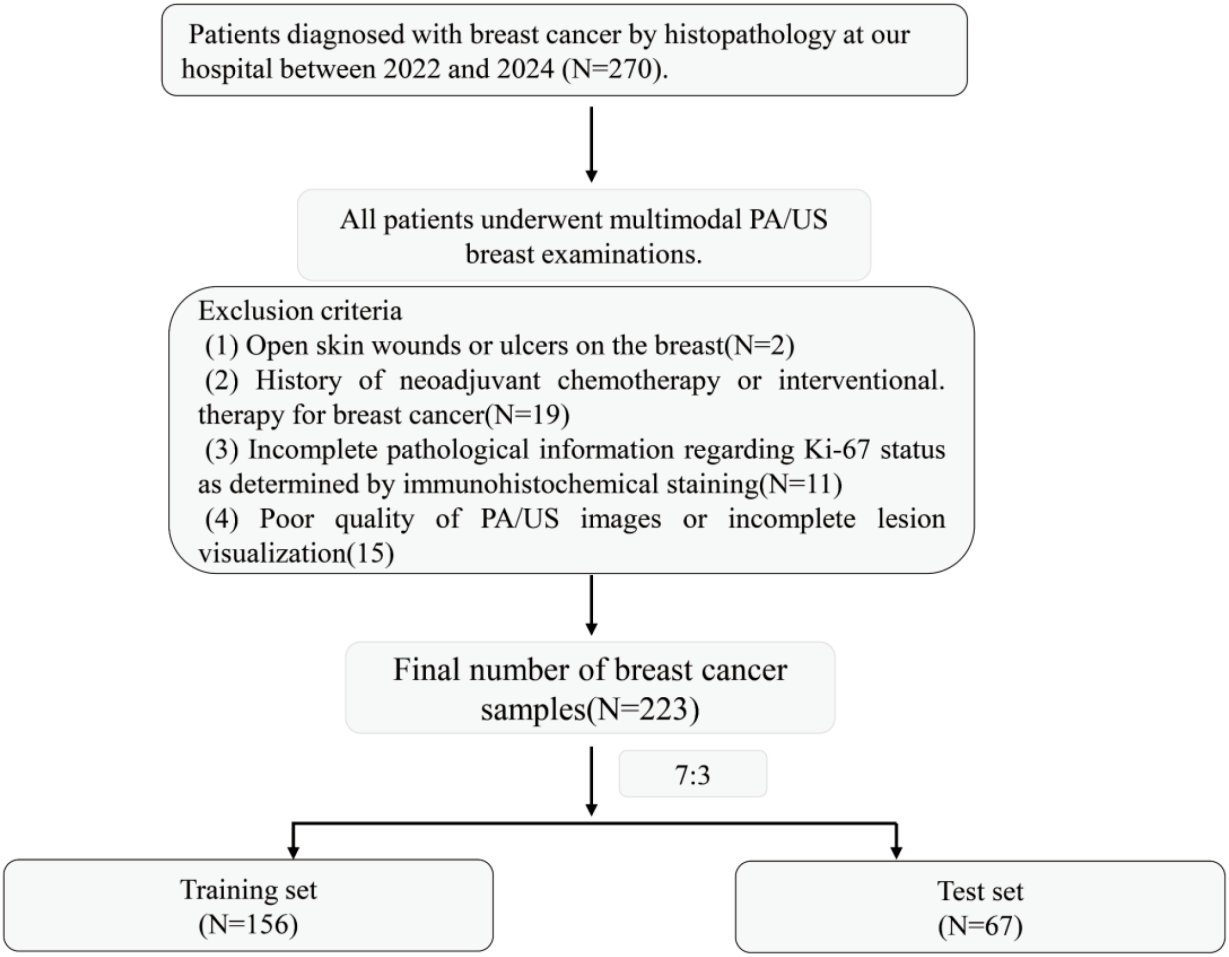
Flowchart of patient inclusion and exclusion criteria.

Between 2022 and 2024, we consecutively enrolled patients with pathologically confirmed BC who underwent multimodal PA/US breast imaging followed by surgery. The exclusion criteria were as follows: (1) open skin wounds or ulceration on the breast, (2) previous neoadjuvant chemotherapy (NAC) or interventional therapy for BC, (3) incomplete pathological information regarding Ki-67 status based on IHC analysis, and (4) poor PA/US image quality or incomplete lesion visualization. A flowchart depicting patient selection is shown in **Figure 1**. According to these criteria, 223 patients with BC were included. PA/US image data, along with clinical and histopathological information, were collected for each patient. All cases were randomly divided into a training set and a testing set at a ratio of 7:3. This proportion was chosen because it is commonly used in machine learning studies and allows adequate data for model training while preserving an independent cohort for testing. The training set was used for model building, parameter tuning and optimization, while the testing set was used to evaluate final model performance.

### 2.2 Pathological analysis

Standard IHC methods were used to determine histologic type, ER, PR, HER2 and Ki-67 expression. ER and PR positivity was defined as nuclear staining in at least one percent of tumor cells, while staining below 1% was considered negative. HER2 status was evaluated based on IHC score, where 0 or 1+ was considered negative and 3+ was considered positive. For equivocal 2+ cases, fluorescence *in situ* hybridization (FISH) was performed to confirm HER2 amplification. Ki-67 expression was quantified according to the St. Gallen consensus (24), where high expression was defined as at least 14% positive nuclei and low expression as less than 14%. This threshold has been widely used in previous breast cancer studies and allows for comparability across studies.

### 2.3 Multimodal PA/US image acquisition

**Figure 2:**
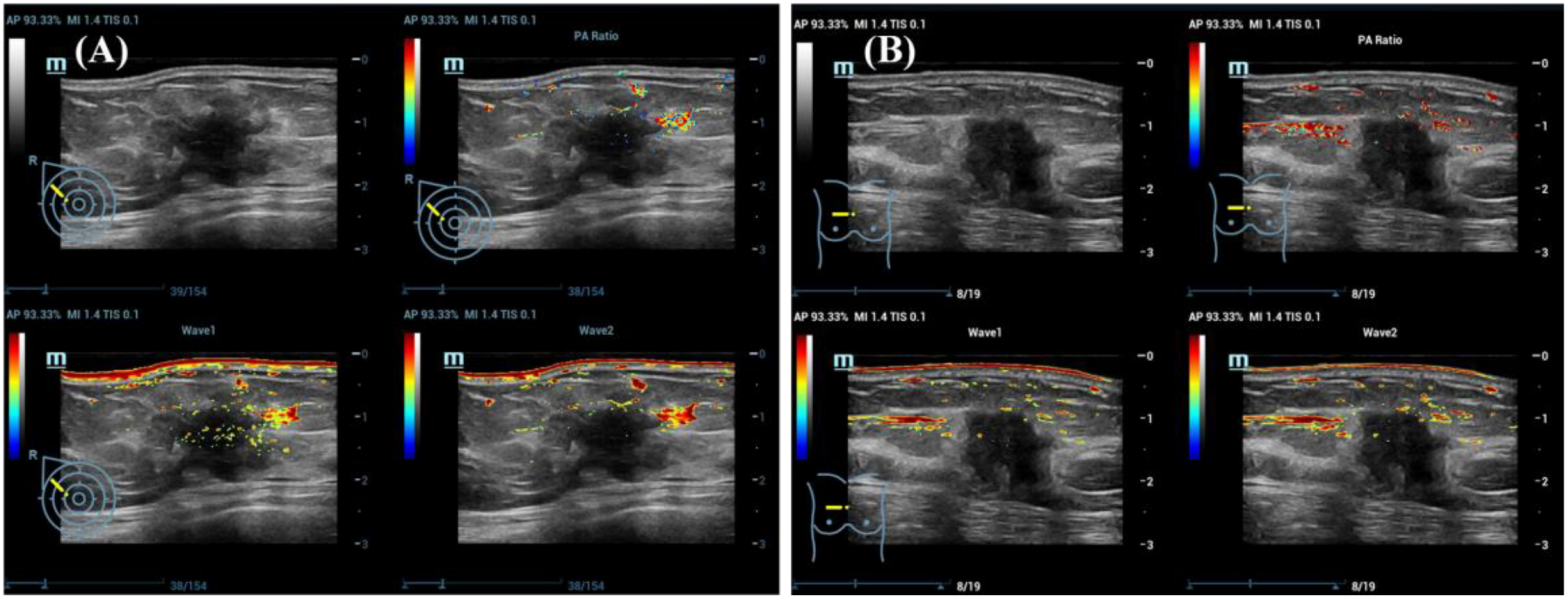
Multimodal photoacoustic/ultrasound (PA/US) images of breast carcinomas with varying Ki-67 expression levels. Each panel displays fused PA/US images and greyscale ultrasound (GSUS). **(A)** Demonstrates a typical case of Ki-67 low-expression breast cancer exhibiting irregular shape, non-parallel orientation, indistinct margins, lobulation and spiculation, low internal echoes, absence of posterior acoustic shadowing, and structural distortion. The PA/US fusion image reveals intense red pseudocolor signals within and surrounding the tumor; **(B)** Presents a representative case of Ki-67-high expression, exhibiting irregular shape, non-parallel orientation, indistinct margins, fine lobulation and angular features, low internal echoes, and posterior acoustic attenuation. The PA/US fusion image reveals moderate red pseudocolor signal predominantly located at the tumor periphery; in multimodal mode, the real-time imaging screen is divided into four sections. Within the multimodal display interface, the real-time imaging screen is divided into four quadrants: the upper left quadrant presents the standard greyscale ultrasound image. The bottom two quadrants display the 750 nm (Wave 1) and 830 nm (Wave 2) PA images superimposed over the GSUS image. The upper right quadrant presents the pseudo-color blood oxygen saturation (SO_2_) distribution map generated by the composite signal of the 750 nm and 830 nm PA images.

All examinations were performed using a multimodal PA/US system equipped with an L9–3 linear probe (Resona 7, Mindray, China) integrated with an optical parametric oscillator laser (Innolas Laser GmbH, Germany). Patients were examined in the supine position, and lesions were initially localized using grayscale ultrasound. Dual-wavelength PA imaging at 750 nm and 830 nm was used to assess tissue oxygenation. Blood oxygen saturation (SO_2_) was automatically calculated based on the differential absorption of hemoglobin at these wavelengths. The resulting pseudocolor SO_2_ maps were overlaid on grayscale ultrasound images for further analysis. Details on PA derived SO_2_ computation and validation can be found in the referenced literature (25). Pseudocolor SO_2_ maps were overlaid on GSUS images for visualization (**Figure 2**).

### 2.4 ROI segmentation and radiomics feature extraction

All images were stored in DICOM format. ROI segmentation was independently performed by two radiologists with more than ten years of breast ultrasound experience using ITK SNAP software (version 3.8.0; http://www.itk-snap.org). Disagreements were resolved through discussion. Both radiologists were blinded to clinical and pathological information to ensure objective annotation. Radiomics features were extracted using the pyradiomics Python package (https://pyradiomics.readthedocs.io/en/latest/index.html). A total of 1561 features were extracted from PA/US images for each patient, all defined according to the Image Biomarker Standardization Initiative (IBSI) (26). The raw feature set included shape features, first order statistics and texture features. Texture features were derived from five matrices: gray-level co-occurrence matrix (GLCM), gray-level dependence matrix (GLDM), gray-level run length matrix (GLRLM), gray-level size zone matrix (GLSZM), and neighboring gray tone difference matrix (NGTDM). Wavelet filtered features were also generated to expand representation of first order and texture characteristics.

### 2.5 Radiomics feature selection

To ensure the robustness and relevance of the extracted radiomics features, a multi-step feature selection strategy was applied. First, feature reproducibility was assessed using intraclass correlation coefficients (ICC) based on 30 randomly selected PA/US images. Only features with ICC greater than 0.80 were retained for further analysis. Next, all retained features were standardized using z-score normalization. Univariate analysis was then performed using t-tests or Mann–Whitney U tests, and features with p < 0.05 were considered statistically significant. To reduce redundancy, Spearman correlation analysis was conducted. When the correlation coefficient between two features exceeded 0.9, one of the features was removed using a recursive elimination strategy. Finally, least absolute shrinkage and selection operator (LASSO) regression with ten-fold cross-validation was applied to further select the most informative features. Features with nonzero coefficients at the optimal lambda were retained for model construction.

### 2.6 Model construction and performance evaluation

**Figure 3:**
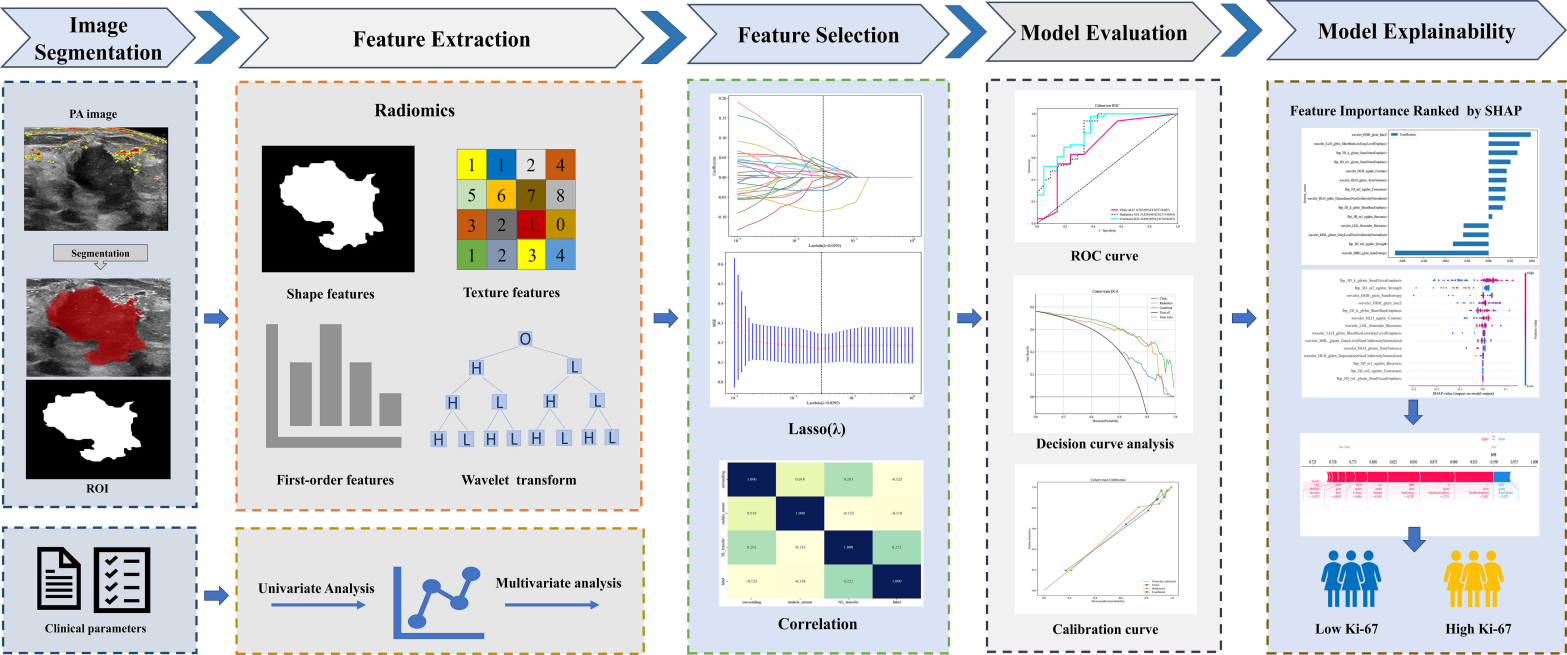
Overall workflow of this study. PA, photoacoustic; ROI, region of interest; LASSO, least absolute shrinkage and selection operator; ROC, receiver operating characteristic curve; SHAP, Shapley additive explanations.

To achieve optimal diagnostic performance, the selected radiomics features were combined with multiple machine learning classifiers, and the classifier with the best overall performance was used to build the radiomics model. Ten commonly used classifiers were evaluated, including LR, SVM, RandomForest, ExtraTrees, XGBoost, NaiveBayes, LightGBM, GradientBoosting, AdaBoost and MLP. Univariate and multivariate logistic regression analyses were conducted to identify clinical factors significantly associated with Ki-67 expression. Clinical variables with p less than 0.05 were treated as risk factors and used to build a clinical model. A combined model was then developed by integrating the selected radiomics features and clinical variables. The entire workflow is illustrated in **Figure 3**. Model performance was evaluated using AUC, accuracy, sensitivity, specificity, NPV and PPV derived from ROC analysis. Calibration curves were generated to assess model agreement, and DCA was performed to examine net clinical benefit across a range of threshold probabilities.

### 2.7 SHAP explainability analysis

To enhance transparency and clinical interpretability of the radiomics model, SHAP (Shapley Additive exPlanations) analysis was used to quantify the contribution of each selected feature to Ki-67 prediction. SHAP, rooted in cooperative game theory, calculates the marginal contribution of individual features to model output and provides both global and local explanations (27). Following official SHAP recommendations, we calculated and visualized SHAP values for the best performing classifier. Summary bar plots and bee swarm plots were generated to illustrate the overall impact and direction of feature effects, while waterfall plots and force plots explained how specific features contributed to individual predictions.

### 2.8 Statistical analysis

Statistical analyses were conducted using R software (version 3.6.3, https://www.r-project.org) and Python (version 3.5.6, http://www.python.org). Continuous variables were compared using t tests or Mann Whitney U tests, and categorical variables were analyzed using chi square tests or Fisher exact tests. A two-sided p value < 0.05 was considered statistically significant.

## 3 Results

### 3.1 Clinicopathological characteristics

A total of 223 patients with BC were included in this study. Based on pathological assessment, 45 patients had low Ki-67 expression and 178 had high expression. The main clinicopathological characteristics, including age, BMI, menopausal status, BI-RADS category, maximum tumor diameter, morphology, margin, echogenicity and posterior acoustic features, internal and external vascularity, ultrasound reported axillary lymph node (ALN) status, ER, PR, HER2, Ki-67 and molecular subtype, are summarized in **Table 1**. When comparing clinicopathological variables between the training and testing sets, no statistically significant differences were observed (p greater than 0.05).

**Table 1:** Clinical characteristics comparison between training and test sets.

Variables	Total (n=223)	Training set (n=156)	Test set (n=67)	P value
Age	49.27 [42.50;58.00]	49.31 [42.00;57.25]	49.16 [45.00;59.00]	0.917
BMI	23.10 [20.30;25.50]	23.15 [19.80;25.50]	22.60 [21.00;25.50]	0.843
Menopause				0.589
No	111 (49.78%)	80 (51.28%)	31 (46.27%)	
Yes	112 (50.22%)	76 (48.72%)	36 (53.73%)	
BI-RADS				0.938
4a	39 (17.49%)	26 (16.67%)	13 (19.40%)	
4b	60 (26.91%)	43 (27.56%)	17 (25.37%)	
4c	106 (47.53%)	75 (48.08%)	31 (46.27%)	
5	18 (8.07%)	12 (7.69%)	6 (8.96%)	
SO_2_	86.85 [83.04;89.87]	86.96 [83.35;90.12]	86.32 [82.55;89.30]	0.254
Maximum diameter	21.00 [16.00;31.50]	22.00 [16.75;32.00]	20.00 [15.00;28.00]	0.452
Shape				0.766
Regular	20 (9.22%)	15 (9.93%)	5 (7.58%)	
Irregular	197 (90.78%)	136 (90.07%)	61 (92.42%)	
Margin				0.737
Circumscribed	50(28.57%)	36(25.93%)	14(29.75%)	
Indistinct	125(71.43%)	85(74.07%)	40(70.25%)	
Intratumoral blood flow				0.479
No detectable blood	14 (6.45%)	10 (6.62%)	4 (6.06%)	
Hypo vascular Blood	64 (29.49%)	48 (31.79%)	16 (24.24%)	
Hyper vascular Blood	61 (28.11%)	38 (25.17%)	23 (34.85%)	
Rich Blood	78 (35.94%)	55 (36.42%)	23 (34.85%)	
Peritumoral blood flow				0.520
No detectable blood	25 (11.52%)	18 (11.92%)	7 (10.61%)	
Hypo vascular Blood	71 (32.72%)	53 (35.10%)	18 (27.27%)	
Hyper vascular Blood	56 (25.81%)	35 (23.18%)	21 (31.82%)	
Rich Blood	65 (29.95%)	45 (29.80%)	20 (30.30%)	
ALN transfer				0.696
No	154 (70.32%)	110 (71.43%)	44 (67.69%)	
Yes	65 (29.68%)	44 (28.57%)	21 (32.31%)	
ER				0.143
Negative	45 (20.18%)	36 (23.08%)	9 (13.43%)	
Positive	178 (79.82%)	120 (76.92%)	58 (86.57%)	
PR				0.204
Negative	54 (24.22%)	42 (26.92%)	12 (17.91%)	
Positive	169 (75.78%)	114 (73.08%)	55 (82.09%)	
HER2				0.810
Negative	33 (14.80%)	22 (14.10%)	11 (16.42%)	
Positive	190 (85.20%)	134 (85.90%)	56 (83.58%)	
ki-67				0.306
Low	58 (26.01%)	37 (23.72%)	21 (31.34%)	
High	165 (73.99%)	119 (76.28%)	46 (68.66%)	
Molecular subtype				0.464
HER2+	31 (13.90%)	25 (16.03%)	6 (8.96%)	
Luminal B	114 (51.12%)	78 (50.00%)	36 (53.73%)	
Luminal A	68 (30.49%)	45 (28.85%)	23 (34.33%)	
TNBC	10 (4.48%)	8 (5.13%)	2 (2.99%)	

BMI, body mass index; BI-RADS, breast imaging-reporting and data system; ALN, axillary lymph node; ER, estrogen receptor; PR, progesterone receptor; HER2, human epidermal growth factor receptor 2; TNBC, triple-negative breast cancer.

### 3.2 Radiomics feature selection

**Figure 4:**
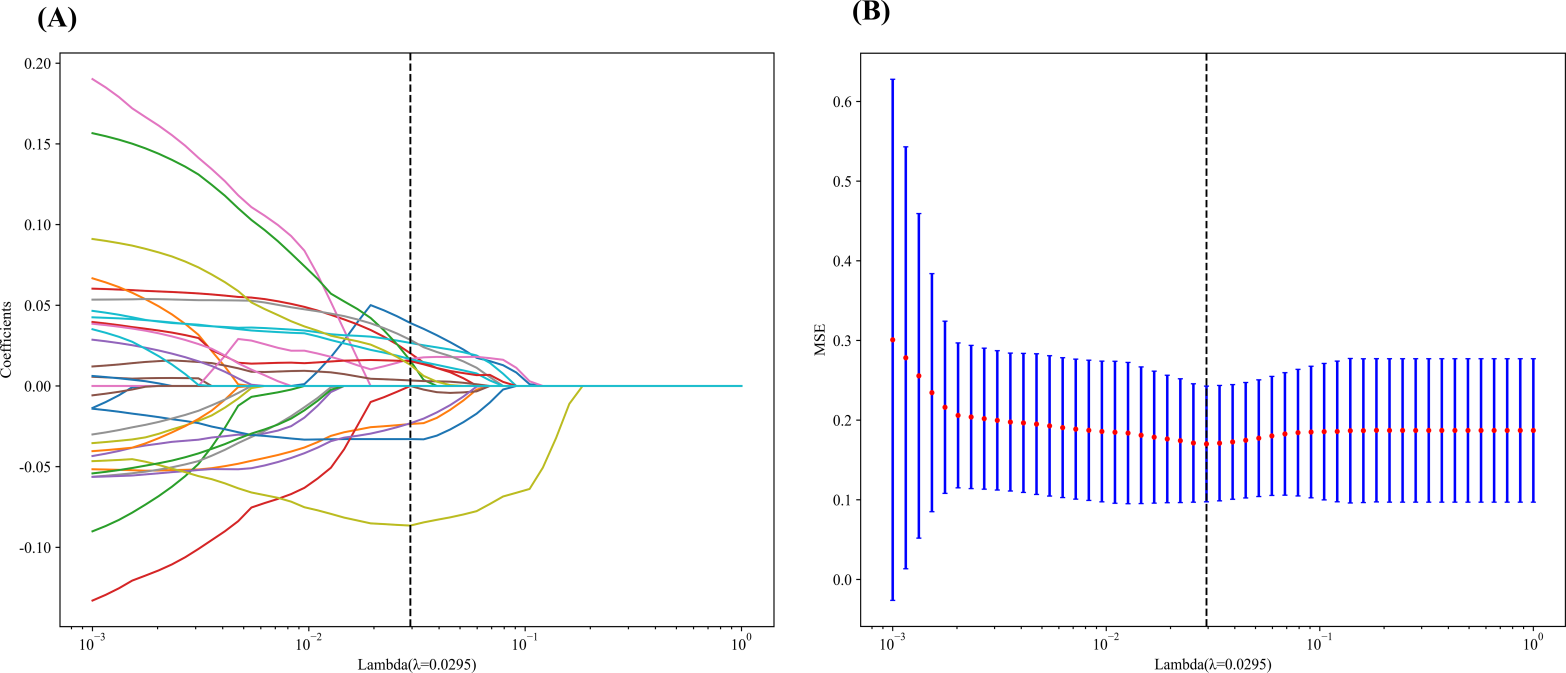
Radiomics feature selection based on the Least Absolute and Selected Operator (LASSO). **(A)** Coefficient distribution plot obtained via the LASSO algorithm using 10-fold cross-validation and **(B)** Mean Squared Error (MSE).

A total of 1561 radiomics features were extracted from each patient’s PA/US images. After z score normalization and removal of features with zero variance or missing values, 1554 features remained. Using t tests or Mann Whitney U tests, 194 features with p values less than 0.05 were identified during univariate filtering. Spearman correlation analysis was then performed to eliminate highly correlated features (r greater than 0.9), and a greedy recursive feature elimination strategy was applied, resulting in a reduced set of 30 features. Finally, LASSO regression with ten-fold cross validation was used to further refine the feature set. Based on the minimum mean squared error criterion, the optimal lambda was determined to be 0.0295 (**Figure 4**). Fourteen features with nonzero coefficients at this lambda were retained as the most representative radiomics predictors.

### 3.3 Construction and evaluation of machine learning classifiers

Using the 14 selected radiomics features, ten widely used machine learning classifiers were constructed to predict Ki-67 expression in BC. These classifiers included LR, SVM, RandomForest, ExtraTrees, XGBoost, NaiveBayes, LightGBM, GradientBoosting, AdaBoost and MLP. Their performance metrics, including AUC, accuracy, sensitivity, specificity, PPV and NPV, are summarized in **Table 2**. In the training set, most ensemble based classifiers such as ExtraTrees, XGBoost, GradientBoosting and AdaBoost demonstrated near perfect discrimination (AUC greater than 0.94), but their performance decreased substantially in the testing set (AUC ranging from 0.645 to 0.689), suggesting potential overfitting. In contrast, RandomForest achieved more stable results across datasets, with AUC values of 0.918 in the training set and 0.826 in the testing set, indicating better generalizability. In the testing cohort, the AUC of the ten classifiers ranged from 0.645 for GradientBoosting to 0.826 for RandomForest. RandomForest achieved the highest AUC and demonstrated strong overall performance, with an accuracy of 0.836, sensitivity of 0.913, specificity of 0.667, PPV of 0.857 and NPV of 0.778. ExtraTrees achieved a comparable AUC of 0.805 but showed lower specificity. SVM, NaiveBayes and MLP showed moderate performance with AUC values between 0.74 and 0.76. LightGBM and XGBoost had lower and less stable performance across datasets. Compared with other classifiers, RandomForest showed more stable performance between the training and testing sets, with less decline in AUC, suggesting better generalizability. It was therefore selected as the optimal model for subsequent analysis.

**Table 2:** Predictive performance of 10 machine learning classifiers in the training and test sets.

Model	Set	AUC (95% CI)	ACC	SEN	SPE	PPV	NPV
LR	train	0.874 (0.805,0.944)	0.846	0.857	0.811	0.936	0.638
LR	test	0.726 (0.581,0.871)	0.791	0.935	0.476	0.796	0.769
NaiveBayes	train	0.830 (0.746,0.914)	0.788	0.798	0.757	0.913	0.538
NaiveBayes	test	0.763 (0.619,0.907)	0.776	0.848	0.619	0.830	0.650
SVM	train	0.837 (0.757,0.917)	0.814	0.832	0.757	0.917	0.583
SVM	test	0.763 (0.629,0.897)	0.821	0.978	0.476	0.804	0.909
RandomForest	train	0.918 (0.865,0.971)	0.808	0.790	0.865	0.949	0.561
RandomForest	test	0.826 (0.711,0.941)	0.836	0.913	0.667	0.857	0.778
ExtraTrees	train	0.972 (0.943,1.000)	0.929	0.933	0.919	0.974	0.810
ExtraTrees	test	0.805 (0.690,0.921)	0.821	0.957	0.524	0.815	0.846
XGBoost	train	0.964 (0.933,0.995)	0.929	0.958	0.838	0.950	0.861
XGBoost	test	0.685 (0.539,0.831)	0.716	0.804	0.524	0.787	0.550
LightGBM	train	0.683 (0.599,0.767)	0.237	0.000	1.000	1.000	0.237
LightGBM	test	0.716 (0.603,0.829)	0.313	0.000	1.000	0.000	0.313
GradientBoosting	train	0.953 (0.913,0.993)	0.904	0.908	0.892	0.964	0.750
GradientBoosting	test	0.645 (0.479,0.812)	0.657	0.674	0.619	0.795	0.464
AdaBoost	train	0.945 (0.912,0.979)	0.853	0.840	0.892	0.962	0.635
AdaBoost	test	0.689 (0.544,0.834)	0.731	0.870	0.429	0.769	0.600
MLP	train	0.875 (0.805,0.945)	0.814	0.807	0.838	0.941	0.574
MLP	test	0.747 (0.604,0.891)	0.806	0.957	0.476	0.800	0.833

AUC, area under the curve; CI, confidence interval; SEN, sensitivity; SPE, specificity; ACC, accuracy; PPV, positive predictive value; NPV, negative predictive value.

### 3.4 Model construction and evaluation

**Figure 5:**
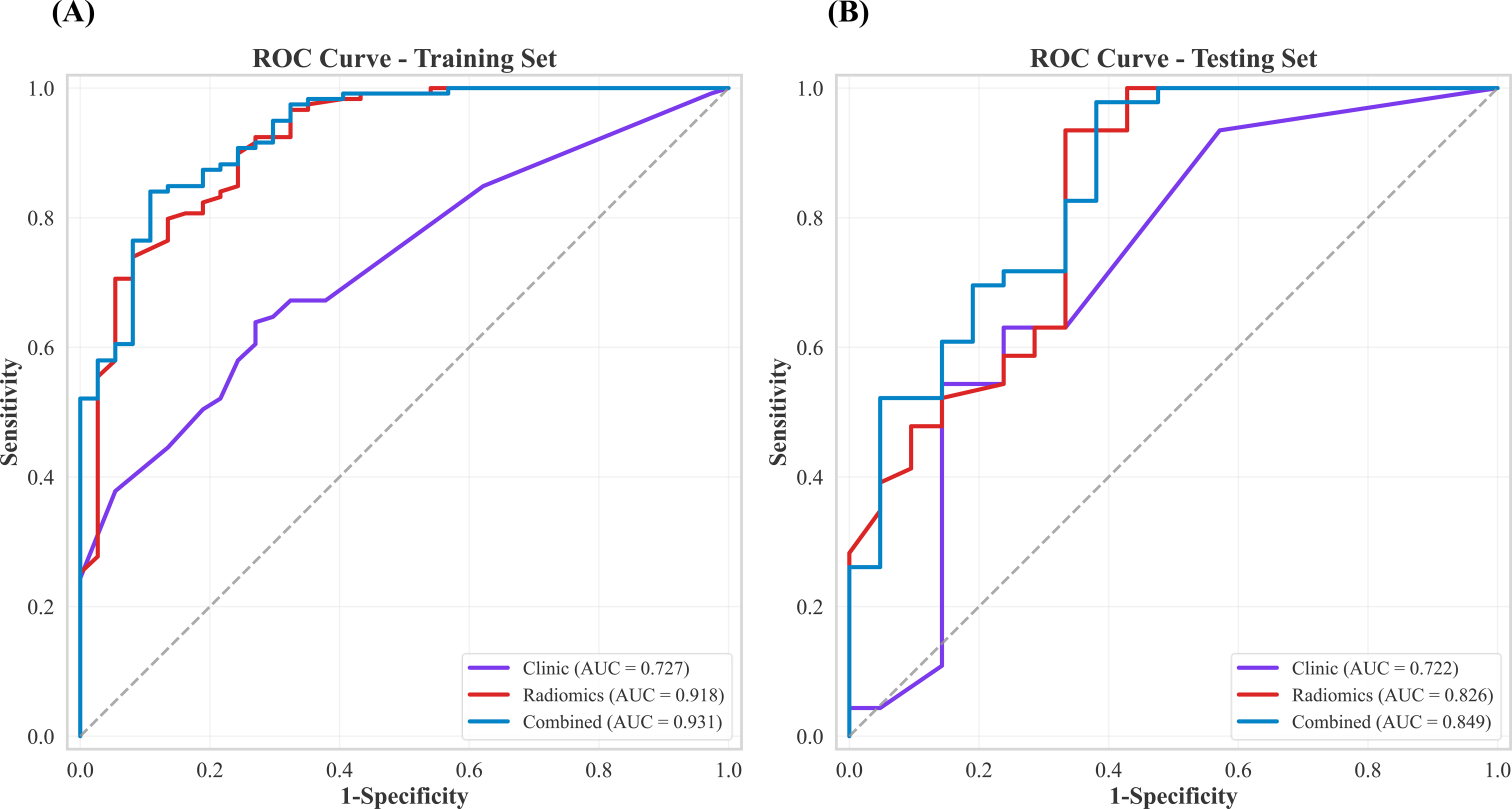
ROC curves for clinic, radiomics, and combined models in the training and testing sets. ROC, receiver operating characteristic curve.

**Figure 6:**
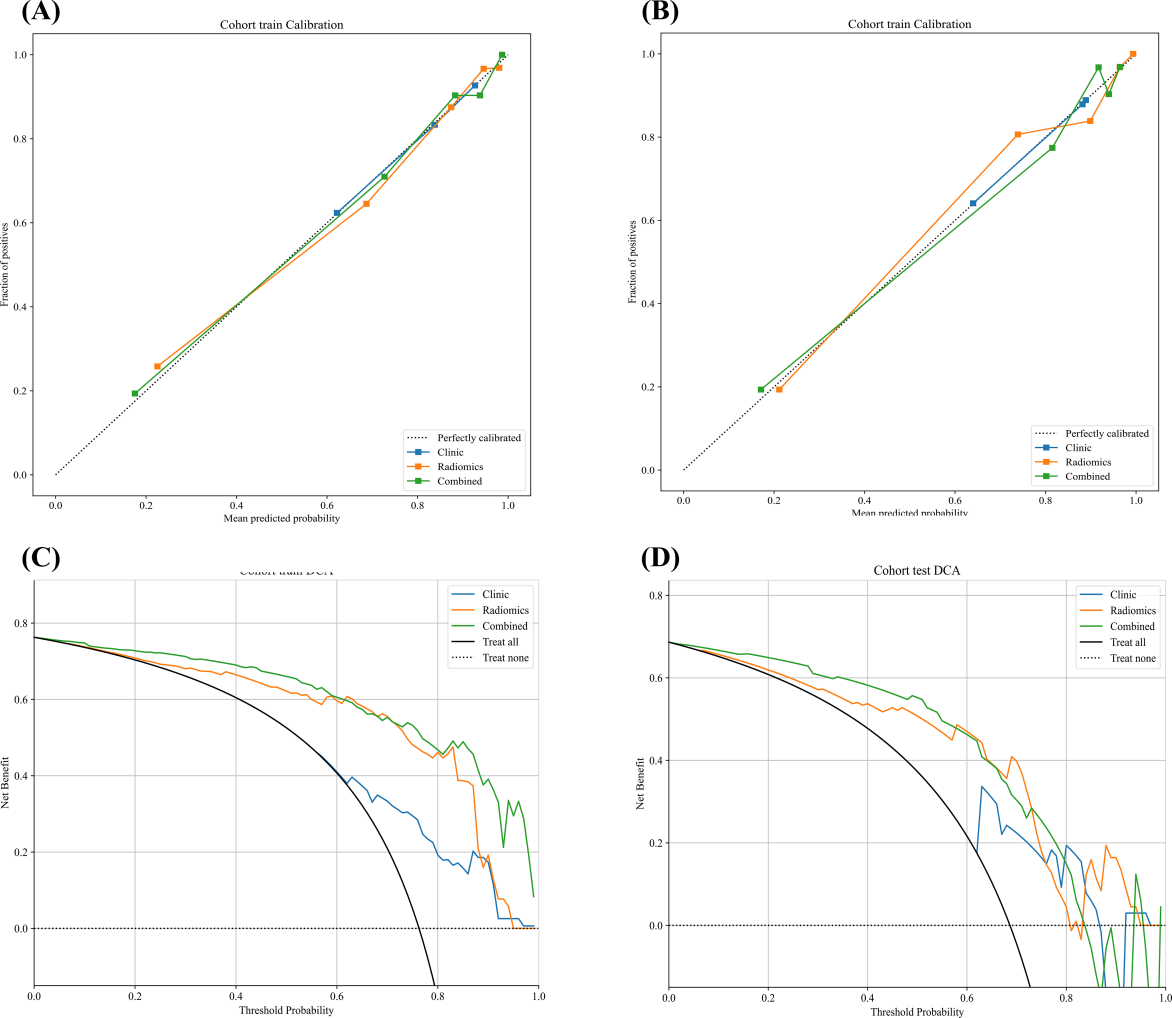
Calibration curves and decision curve analysis (DCA) plots for three models in the training and test sets. **(A, B)** Calibration curves for the clinical model, radiomics model, and combined model in the training and test cohorts. The 45° diagonal line represents perfect calibration. **(C, D)** DCA for the three models in the training and test cohorts, illustrating net benefit at different threshold probabilities.

Using the selected radiomics features, a radiomics model was developed with the RandomForest classifier. Univariate and multivariate logistic regression identified margin, SO_2_ and ALN status as significant predictors (**Table 3**), which were used to construct the clinical model. A combined model integrating both clinical predictors and radiomics features was then developed to further improve performance. As shown in **Table 4** and **Figure 5**, the clinical model, radiomics model and combined model demonstrated progressively improved discrimination. The clinical model achieved AUC values of 0.727 in the training set and 0.722 in the testing set. The radiomics model achieved higher discrimination, with AUCs of 0.918 in the training set and 0.826 in the testing set. When clinical factors and radiomics features were integrated, the combined model achieved further improvement, with AUCs of 0.931 in the training set and 0.849 in the testing set. In the testing set, the combined model achieved the highest sensitivity (0.957) and NPV (0.867), indicating strong ability to correctly identify high Ki-67 expression while accurately ruling out low expression. Compared with the clinical and radiomics models, the combined model also showed better overall performance. Calibration curves (**Figures 6A, B**) demonstrated good agreement between predicted and observed probabilities. DCA (**Figures 6C, D**) further showed that the combined model yielded the highest net benefit across a wide range of threshold probabilities. Together, these findings support the predictive performance and clinical utility of the combined model for preoperative assessment of Ki-67 expression in BC.

**Table 3:** Results of univariate and multivariate analysis. .

Variables	Univariate			Multivariate		
	OR	95%CI	p value	OR	95%CI	p value
SO_2_	1.013	1.010-1.017	p<0.001	1.042	1.009-1.077	0.037
Age	1.023	1.017-1.029	p<0.001	0.997	0.939-1.059	0.937
Maximum diameter	1.046	1.033-1.060	p<0.001	1.006	0.965-1.049	0.820
Peritumoral blood flow	1.436	1.239-1.664	p<0.001	0.427	0.173-1.053	0.121
Intratumoral blood flow	1.482	1.288-1.706	p<0.001	0.829	0.316-2.175	0.749
BI-RADS	1.899	1.565-2.303	p<0.001	1.015	0.583-1.766	0.965
Margin	2.750	1.958-3.861	p<0.001	0.151	0.049-0.471	0.006
Menopausal status	3.222	2.067-5.023	p<0.001	0.767	0.193-3.043	0.751
Shape	3.273	2.358-4.540	p<0.001	2.706	0.808-9.052	0.175
ALN transfer	6.333	3.074-13.040	p<0.001	3.792	1.251-11.496	0.048

BI-RADS, breast imaging-reporting and data system; CI, confidence interval; OR, odds ratio.

**Table 4:** Comparison of the performance of different models based on PA/US images.

Model	Set	AUC(95%CI)	ACC	SEN	SPE	PPV	NPV
Clinic	train	0.727 (0.645,0.810)	0.635	0.605	0.730	0.878	0.365
Clinic	test	0.722 (0.577,0.867)	0.582	0.457	0.857	0.875	0.419
Radiomics	train	0.918 (0.865,0.971)	0.808	0.790	0.865	0.949	0.561
Radiomics	test	0.826 (0.711,0.941)	0.836	0.913	0.667	0.857	0.778
Combined	train	0.931 (0.886,0.975)	0.846	0.832	0.892	0.961	0.623
Combined	test	0.849 (0.745,0.953)	0.851	0.957	0.619	0.846	0.867

AUC, area under the curve; CI, confidence interval; SEN, sensitivity; SPE, specificity; ACC, accuracy; PPV, positive predictive value; NPV, negative predictive value.

### 3.5 SHAP explainability analysis

To improve interpretability and quantify the contribution of each radiomics feature to Ki-67 prediction, SHAP analysis was performed based on the radiomics model. Although the combined model included both clinical and radiomics variables, SHAP analysis focused on the radiomics model to highlight the independent predictive value of imaging features.

**Figure 7:**
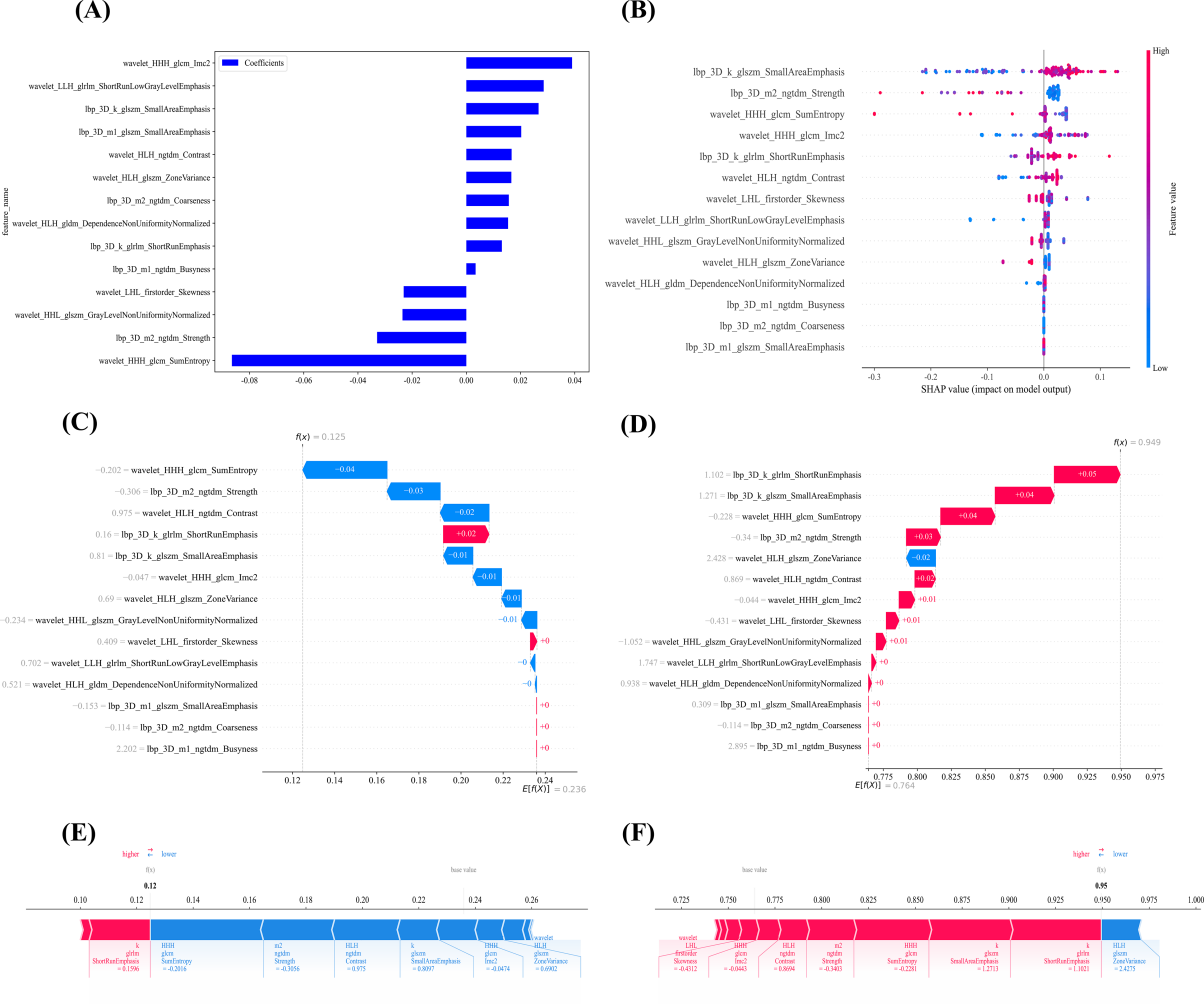
SHAP-based global and local interpretability of the radiomics model for Ki-67 prediction. **(A, B)** present global interpretability of the model. **(A)** The SHAP feature importance plot shows the mean absolute SHAP values of each radiomic feature, where higher values indicate greater contribution to distinguishing low versus high Ki-67 expression. **(B)** The SHAP summary plot illustrates the distribution of feature effects across all patients, with each point representing an individual sample; colors indicate feature values (red for higher values and blue for lower values), and the horizontal axis represents the magnitude of impact on model output. **(C, E)** correspond to a representative patient with low Ki-67 expression, while **(D, F)** correspond to a patient with high Ki-67 expression. **(C, D)** are SHAP waterfall plots showing how individual radiomic features contribute to the final prediction, with blue indicating negative contributions and red indicating positive contributions. **(E, F)** are SHAP force plots that visually demonstrate how the cumulative effects of these features drive the prediction toward low or high Ki-67 expression, respectively. Together, these panels provide both global and patient-level insights into the model’s decision-making process.

The SHAP summary bar plot and bee swarm plot (**Figures 7A,B**) showed that features mainly derived from GLCM and GLSZM had the greatest influence on model output. The top three features were GLCM_Entropy, GLSZM_ZoneEntropy and FirstOrder_Energy. Both GLCM_Entropy and GLSZM_ZoneEntropy were positively associated with high Ki-67 expression, indicating that greater gray level irregularity and structural complexity were linked to increased proliferative activity. In contrast, FirstOrder_Energy contributed negatively, suggesting that higher image uniformity was associated with lower Ki-67 expression. These patterns indicate that intratumoral structural heterogeneity captured by radiomics features plays a key role in predicting tumor proliferation.

Patient-level explanations are shown in **Figures 7C, E** correspond to a patient with low Ki-67 expression, illustrating how multiple radiomics features contributed collectively to a low predicted probability. The waterfall plot highlights the dominant negative contributors, while the force plot shows how negative contributions outweighed positive ones, leading to a low overall probability. In contrast, **Figures 7D, F** represent a patient with high Ki-67 expression. Both the waterfall plot and force plot demonstrate that several texture and intensity related features exerted positive influence, cumulatively pushing the prediction toward a high probability. Together, these paired visualizations demonstrate the local interpretability of the radiomics model and clarify which features, and in which directions, determined individual predictions.

Overall, SHAP analysis quantitatively explained how radiomics features reflecting intratumoral heterogeneity, especially texture features, drive the model’s decision making. This improves the biological interpretability and clinical credibility of the radiomics model and supports its potential role in noninvasive evaluation of tumor proliferation.

## 4 Discussion

Previous studies have confirmed the usefulness of multimodal PA/US imaging in several BC related tasks, including lesion classification (28), axillary lymph node assessment (29), molecular subtype prediction (30) and HER2 characterization (15). However, its role in the noninvasive prediction of Ki-67 expression has been explored far less. To address this gap, we developed and compared three predictive models: a clinical model based on independent predictors identified by logistic regression (tumor margin, SO_2_ and ALN status), a radiomics model derived from PA/US features, and a combined model that integrates both feature sets. Ten machine learning algorithms were evaluated during model development, and RandomForest was selected as the optimal classifier for the radiomics model based on its overall performance. In the testing cohort, the AUCs of the clinical, radiomics and combined models were 0.722, 0.826 and 0.849, respectively. The combined model achieved the highest sensitivity (0.957), indicating excellent ability to detect high Ki-67 expression, and also the highest NPV (0.867), suggesting reliable exclusion of low expression when the prediction was negative. These findings indicate that integrating quantitative PA/US imaging biomarkers with key clinical variables can enhance noninvasive assessment of tumor proliferation and may improve preoperative risk stratification and individualized treatment planning.

Ki-67 is a widely recognized biomarker reflecting tumor proliferative activity. Its expression level is closely associated with breast cancer molecular subtypes, treatment response, and prognosis, making it an important factor in clinical decision-making. IHC remains the clinical standard for Ki-67 assessment, yet it has inherent limitations in the context of aggressive and heterogeneous BC, where sampling only part of the lesion can lead to bias (5, 31). These limitations constrain the role of IHC in comprehensive, noninvasive preoperative evaluation. MRI has been investigated as an imaging based surrogate of proliferative activity, but its routine use is restricted by cost, scan time and accessibility (9, 32). In contrast, PA/US offers a more practical alternative, with real time acquisition, wide availability and the ability to provide functional information. In particular, PA derived SO_2_ reflects tumor oxygenation and perfusion, parameters that are biologically linked to proliferation (33, 34) and therefore to Ki-67. In this study, we combined PA/US imaging with radiomics to quantitatively extract morphological and functional imaging features of the lesions. This framework may serve as a complementary noninvasive tool for preoperative assessment of tumor proliferation and may help support individualized treatment decisions.

The feasibility of using radiomics to predict Ki-67 expression in BC has been demonstrated previously, although most work has been based on MRI, with reported AUCs typically in the range of about 0.64 to 0.74 (35, 36). These values suggest moderate discrimination but also indicate room for improvement. Moreover, MRI examinations are costly, time consuming and less accessible in routine practice, and oxygenation related information is usually captured only indirectly. By contrast, PA combined with US can acquire structural images and oxygenation related functional signals such as SO_2_ on a conventional ultrasound platform, providing complementary clues that are closely related to proliferation (13). In our study, tumor margin morphology, SO2 and ALN status were associated with high Ki-67 expression. The clinical model based on these variables achieved an AUC of 0.722 in the testing set, whereas the radiomics model based solely on PA/US features reached an AUC of 0.826. This comparison suggests that although clinical variables are informative, their discriminative power is limited. Incorporating PA derived quantitative phenotypes allows the model to better capture structural and functional signatures linked to proliferative activity.

Previous studies that used ultrasound and clinicopathologic features to build nomograms for predicting Ki-67 in BC reported moderate performance. Liu et al. reported an AUC of 0.770 (37), and Wu et al. reported an AUC of 0.808 (38). In comparison, our combined model that integrates clinical variables with PA/US radiomics features achieved an AUC of 0.849 in the testing set and showed the best sensitivity and NPV. This advantage may partly arise from the complementary contributions of PA derived tissue oxygen saturation and texture phenotypes, enabling the model to capture both microvascular oxygenation and tissue heterogeneity. From a clinical decision perspective, DCA showed that across a broad range of threshold probabilities, the combined model consistently yielded higher net benefit than the clinical only and radiomics only models, as well as the treat all and treat none strategies. Calibration analysis further demonstrated good agreement between predicted probabilities and observed outcomes, suggesting reliable probability estimates in this cohort (39). Taken together, these findings support PA/US radiomics as a useful adjunct for preoperative Ki-67 assessment, with both discriminative and decision-making value.

Another strength of this study is the incorporation of the SHAP framework to enhance model interpretability. Machine learning models are often criticized for their black box nature (40), and transparent explanations are essential for clinical adoption. By combining the RandomForest classifier with SHAP analysis, our model not only achieved strong predictive performance but also provided biologically meaningful insight into its decision process. At the global level, SHAP analysis showed that texture based radiomics features reflecting intratumoral heterogeneity played a dominant role in predicting Ki-67 expression. This finding suggests that spatial irregularity and structural complexity of the tumor, quantified by gray level based features, are closely linked to proliferative activity. This is consistent with the biologically plausible concept that tumors with more disordered microstructural patterns tend to have higher proliferative potential. At the individual level, SHAP visualizations illustrated how different image derived features jointly shape the predicted probability for each patient. Such interpretability allows clinicians to trace model decisions back to specific quantitative imaging biomarkers, transforming the model from a purely statistical tool into a clinically interpretable decision aid. Overall, the SHAP based framework connects statistical learning with pathophysiological understanding. By clarifying the internal logic of the radiomics model, it enhances trust and transparency and supports the integration of PA/US radiomics into clinical workflows for noninvasive assessment of tumor proliferation.

Despite these encouraging results, several limitations should be acknowledged. First, this was a single center study, which may limit the generalizability of the findings. PA/US remains a relatively new technology and is not yet widely implemented, which makes multi center data collection challenging. To mitigate selection bias, all imaging and pathological assessments were performed using standardized acquisition protocols. Future work with larger multi center cohorts is needed to further validate the robustness of the proposed models. Second, tumor ROIs were manually delineated by experienced radiologists. Although manual segmentation may introduce a degree of subjectivity, we evaluated interobserver agreement using ICC and retained only features with ICC values greater than 0.80. In future studies, deep learning based automatic segmentation methods may improve reproducibility and facilitate broader clinical application. Third, radiomics features were extracted from two-dimensional PA/US images, which may not fully capture the three-dimensional spatial heterogeneity of breast tumors. At present, two-dimensional imaging remains the clinical standard given current technical constraints. However, advances in three-dimensional PA/US imaging may enable more comprehensive characterization of tumor biology and further enhance predictive performance.

## 5 Conclusion

This study demonstrates that integrating radiomics analysis with PA/US imaging enables noninvasive and quantitative assessment of tumor proliferative activity in BC. By extracting radiomics features that reflect intratumoral heterogeneity and combining them with clinical variables, the proposed models effectively predicted Ki-67 expression and outperformed single modality approaches. The incorporation of SHAP based explainability further clarified the biological relevance of key imaging biomarkers and improved the transparency and clinical credibility of the model. Taken together, these findings highlight the potential of PA/US radiomics as a valuable complement to conventional IHC assessment, providing a reproducible and objective imaging strategy for evaluating tumor proliferation. With further validation in larger and multi center cohorts, this approach may offer a promising framework to support individualized treatment planning and prognostic assessment in patients with BC.

## Data Availability

The datasets generated and/or analyzed during the current study are not publicly available due to patient privacy and institutional restrictions but are available from the corresponding author on reasonable request. Requests will be considered in accordance with ethical, legal, and non-commercial research requirements.
